# Family functioning and life satisfaction among female university students during COVID-19 outbreak: the mediating role of hope and resilience

**DOI:** 10.1186/s12905-022-02103-3

**Published:** 2022-12-05

**Authors:** Salman Zarei, Khadijeh Fooladvand

**Affiliations:** Psychology Department, Lorestan University, Khorramabad, Iran

**Keywords:** Family functioning, Life satisfaction, Hope, Resilience, COVID-19, Female university students

## Abstract

**Background:**

Literature review has showed that family functioning is positively associated with satisfaction with life. However, the internal mechanisms of this relationship are still unclear, especially during the COVID-19. This study examined the mediating role of hope and resilience in the association between family functioning and life satisfaction of female university students in COVID-19 pandemics.

**Methods:**

A cross-sectional online study was done throughout the fifth wave of the COVID-19 outbreak in Iran. A total of 480 female students were recruited from Lorestan University. Data was collected by the State Hope Scale, Satisfaction with Life Scale, Family APGAR Index, and Connor–Davidson Resilience Scale. Data analysis were based on structural equation modeling.

**Results:**

Family functioning exerted a significant direct effect on hope, on resilience and on life satisfaction. Also, according to the findings, hope and resilience pose a significant mediating effect in the association between family functioning and life satisfaction.

**Conclusion:**

This study provides a better perspective regarding the protective role of hope, family functioning, and resilience on life satisfaction among female university students during the COVID-19 pandemic. Training of such skills is needed to increase life satisfaction in female university students.

## Background

The Coronavirus Disease 2019 (COVID-19) pandemic has strongly affected public health globally [1]. The surge of people infected with COVID-19 and the strict public health regulations (e.g., quarantine, physical distancing, shouting down colleges, and prohibiting large student gatherings) have increased the COVID-19 pandemic effect, decreasing people’s life satisfaction [2, 3]. Due to this pandemic, face-to-face classes were canceled in over 194 countries, influencing over 91% of students [4, 5]. As a result, undergraduates faced more stressors due to the protection and prevention measures affecting education, as in-person activities were interrupted and virtual classrooms using the internet were developed worldwide, increasing psychological distress symptoms (e.g., stress and anxiety) and decreasing life satisfaction [2, 6].

Life satisfaction usually indicates a judgmental process where people holistically assess the status of their lives according to their distinct criteria [7]. Life satisfaction is essential to successful life adaptation, with advantages for longevity, health, and social interactions [8, 9]. Resilience and social support [10], hope and optimism [11], healthy family relationships, quality of family context, and positive family climate [12–14] can predict increased life satisfaction, while depression, stress, anxiety, and COVID-19 uncertainty reduce life satisfaction [15–17]. Studies performed during the COVID-19 pandemic showed a significant decrease in well-being and life satisfaction [18, 19]. Similarly, studies from different countries showed that the COVID-19 pandemic reduced life satisfaction in university students [20–22]. A cross-sectional study reported that university students’ life satisfaction during the COVID-19 pandemic was below moderate [23]. It was confirmed in another study that could establish a link between more fear of COVID-19 and lower life satisfaction in university students [2].

The impacts of crises are never gender-neutral, and COVID-19 is no exception. In this line, many recent studies have reported that the female gender was related to lower life satisfaction and higher stress, anxiety, and depression during the COVID-19 pandemic [19, 24–26]. One cross-sectional study showed that female university students experienced significantly higher anxiety levels than their male counterparts during the COVID-19 pandemic [20]. Similarly, another cross-sectional study found that the COVID-19 pandemic negatively impacted females’ life quality and mental health more than males’ [27]. Therefore, considering evidence that COVID-19 particularly impacts females, the present study focused on female students.

Family functioning may be fundamental in increasing or decreasing life satisfaction among female students [28]. Family functioning is the quality of interactions between family members, as well as family structure, relationships, support for each other, and expectations [29]. Also, based on Olson’s Circumplex Model of Family Systems [30], the family function is divided into three dimensions: Adaptability (how the family system can balance change and stability), cohesion (the ability to preserve powerful emotional bonds between family members, and mutual communication (that increases family cohesion, adaptability, and flexibility) [31]. This model is a theoretical framework focusing on the relationship between life satisfaction and family balance. According to this model, a balanced family is characterized by too few interactions or much consensus in the family, which positively influences an individual’s life satisfaction [32]. Some recent studies have confirmed this notion. For instance, some research showed a strong association between a high level of life satisfaction and family function [32]. Also, Huang et al. [33] reported an association between cohesive family relationships and life satisfaction in postgraduate medical students. Accordingly, we proposed our first hypothesis as follows:

### Hypothesis 1

A significantly positive correlation exists between female university students’ life satisfaction and family functioning.

Satisfaction with life is influenced by family functioning and psychological resources [34, 35]. According to the Broaden-and-Build Theory, an individual’s adaptation to society can be promoted by positive emotions through creating sustainable psychological resources, which can finally predict their life satisfaction judgments [36]. Positive psychological resources are helpful for adaptation to changing demands and improving emotional stability in the face of crisis, like a pandemic, hence enhancing life satisfaction [37]. Hope, as a positive psychological resource, helps reshape self-confidence and improve capability, and enables people to follow a better state (through addressing dilemmas). It includes physiology, psychology, and sociology, allowing people to establish positive values and beliefs and participate in more pro-social behaviors, which let people overcome problems. People with more hope agree more with their goals, preserve higher motivation in following them, and tend to be satisfied with their achievements in life [38].

Most cross-sectional studies have suggested that hope strongly correlates with higher life satisfaction [39, 40]. Also, longitudinal studies have indicated that hope is a crucial predictor of later life satisfaction following the control of initial life satisfaction [41]. Consistent with Olson’s Circumplex Model of Family Systems [30] and Broaden-and-Build Theory [36], the family function may promote life satisfaction via hope, which is considered an enhancing factor. If the degree to which a person regards the quality of interactions among family members (family functioning) as important is higher, their ability to see a desirable outcome as a genuine possibility (hope) is higher [42, 43]. In this context, it can be assumed that hope and family functioning protect against negative emotions during the COVID-19 pandemic among female students and improve their life satisfaction [44]. In addition, hope can mediate the relationship between life satisfaction and family function [11, 45]. Accordingly, we proposed our second hypothesis:

### Hypothesis 2

Hope plays a mediating role between family functioning and female university students’ life satisfaction.

As discussed, the association between life satisfaction and family functioning may go beyond the direct link, and other indirect factors may further explain this relationship. Based on Olson’s Circumplex Model of Family Systems [30] and Kumpfer Resilience Model [46], we proposes resilience as a probable mediator between family functioning and life satisfaction among female university students. Resilience is the dynamic process and capacity of adaptation to overcome adversity and stress while preserving normal physical and psychological functioning [47]. It plays a role in reaching a higher level of balance or returning to the initial balance, resulting in life satisfaction and positive compatibility [48]. Resilient female students are assertive, have practical social skills, and can control their emotions appropriately. Less resilient female students cannot cope with traumatic situations and are at risk of developing depression and anxiety, which may affect their life satisfaction [49].

Studies have verified that resilience is positively associated with life satisfaction [50, 51]. For example, Prayag et al. [52] examined the effects of resilience on life satisfaction and confirmed its positive effects on employees. Accordingly, Baykal [53] found that psychological resilience can promote life satisfaction during the COVID-19 pandemic. Moreover, a cross-sectional study of 1,032 college students during the COVID-19 outbreak showed that college students, especially female students with higher levels of emotional resilience, reported higher life satisfaction [54]. On the other hand, empirical studies showed that resilience is positively associated with family functioning [55, 56]. Moreover, some studies have shown that the components of resilience are directly linked to family functioning, especially the family cohesion component [57, 58], making resilience a vital factor in explaining family functioning. Accordingly, life satisfaction can result from healthy family functioning and resilience. Therefore, we proposed our third hypothesis:

### Hypothesis 3

Resilience has a mediating role between family functioning and female university students’ life satisfaction.

Altogether, higher life satisfaction can predict the mental state of individuals [59] and improve female college students’ academic well-being and performance [60], and lower life satisfaction can predict mental dysfunction [61]. Therefore, improving life satisfaction is critical for college students to adapt to college and grow up healthily. Hope and resilience have been linked to the core components of family functioning and life satisfaction; however, they have been less investigated as mediators and have not been studied concerning life satisfaction in Iranian female university students during the COVID-19 pandemic. Accordingly, this research investigated the mediating role of resilience and hope in the relationship between family functioning and life satisfaction among female university students.

## Methods

This cross-sectional online survey study was done throughout the fifth wave of the COVID-19 outbreak, from July to December 2021, in Iran. A total of 480 female students were recruited from Lorestan University. No consensus is available regarding the sample size of path analysis studies and the minimum sample size of 200 cases has been recommended [62].The eligibility criterions included: (1) being a female (2) being an undergraduate student, (3) being willing to take part in the study. A history of mental disorders was the exclusion criterion. The survey was generated and was provided to in all universities via Google Forms. Students were received invitations to answer the online questionnaire via e-mail and social media (SoroushPlus Messenger, Whatsapp, Telegram and Facebook,). Prior to answering the formal survey, the participants signed informed consent and then completed the questionnaire. Also, participants could leave the study at any time and their data kept confidential. Seventeen cases missed more than 50% of items and were not included for further analyses. Thus, the valid response rate of this study was 96.45%. Among the 463 remaining participants, there were 33 (7.1%) freshman, 102 (22.1%) sophomore, 138 (29.8%) junior, 190 (41%) senior, aged between 18 and 26 (M = 22.54, SD = 1.83).

### Research instruments

Data were gathered by a self-report, structured online survey, which assessed socio-demographic, such as age and grades and also four psychometric measures.

### Satisfaction with life scale (SWLS)

The SWLS consists of a five-item global assessment of life satisfaction, which is a seven-point Likert-type response scale from 1 = strongly disagree to 7 = strongly agree. The total score is 5–35, with higher scores indicating high life satisfaction. Diener et al. [63] compared this scale with others to indicate its validity. They administered 13 external measures and applied two samples besides the SWLS. A moderate strong correlation was found between the SWLS and other tools (except for AIM), and outcomes ranged from 0.50 to 0.75 for different samples and measures. Its validity and reliability have been confirmed using the SWLS in studies on Iranian university students [64]. In our research, its Persian version showed good internal consistency (Cronbach’α = 0.88).

### Family APGAR iIndex

The APAGAR was developed to assess family functioning and includes five items representing partnership, adaptability, growth, resolve and affection and are evaluated regarding the extent to which the respondent agrees with it ranging from 0 (hardly ever) to 2 (almost always). The total score is from zero to 10, with a score smaller than three indicating poor family functioning, and a score more than seven indicating good functioning [65]. Its single factor structure (67.52% of the explained variation; KMO measure of sampling adequacy 0.851) was approved [66]. It has been extensively applied in Iranian university students’ samples and showed great validity and reliability [67]. In our research, its Persian version showed good internal consistency (Cronbach’α = 0.89).

### State hope scale (SHS)

The SHS has six items assessing agency thinking (“I meet the goals that I set for myself”) and pathways thinking (“I can think of many ways to get out of a jam”), ranging from 1 (completely disagree) to 8 (completely agree). Even- and odd-numbered items are summed to obtain an agency subscale score and a pathways subscale, respectively. All items are summed to calculate total state hope score. Its total score is from 6 to 48, with scores indicating a higher degree of sense of hope. Snyder et al. [68] reported that the SHS is internally consistent and reflects the theorized agency and pathways components. Its Iranian version has been extensively applied with good validity and reliability among students [69]. In our research, its Persian version showed good internal consistency (Cronbach’α = 0.91).

### Connor–Davidson resilience scale (CD-RISC)

This scale with 25 items is scored on a five-point Likert scale ranging from 0 (Not true at all) to 4 (True nearly all the time) and the total score is from 0 to 100. Higher scores indicate higher resilience levels. This scale has five factors of Personal competence, Spiritual influences, Tolerance of negative emotions, Positive acceptance of change, and Perceived control. It has been validated in many cultures and countries. Exploratory factor analysis confirm the 5 factor structure of this scale [70]. Its Iranian version is a reliable and valid scale [71]. In our research, it showed good internal consistency with Cronbach’s alpha coefficient 0.92.

### Statistical analysis

SPSS 23.0 analyzed the data. Initially, the descriptive statistics of the socio-demographic features in nurses were performed, followed by. Pearson’s correlation coefficient to assess the association among hope, family functioning, life satisfaction and resilience. Then, AMOS 21.0 was applied to assess the models according to the fit indices of chi-square (*x*^2^), root-mean square error of approximation (RMSEA) Tucker–Lewis index (TLI), comparative fit index (CFI), and standardized root-mean square residual (SRMR). The non-significant chi-square indicated good fit of model-data. The general cutoffs for accepting a model are equal to or greater than 0.90 for the CFI and TLI, and less than 0.08 for the SRMR and RMSEA [72].

## Results

The findings revealed that the statistical testing power of each of the paths of the model was over 0.90 after the sample size reached 430. Thus, the researchers concluded that the sample size of the present study was sufficient. Due to the self-report nature of the data, there was a possibility of common method bias. Therefore, the Harman’s single-factor test was conducted [73]. The results showed that there were six factors with characteristic roots greater than 1, and the explanation rate of the first factor was 21.72%, below the threshold of 40%, which indicated that there was no significant common method bias in the study.

Descriptive statistics such as means and standard deviations are presented in Table 1. All study variables including family functioning, hope, resilience and life satisfaction were screened for Skewness and kurtosis to evaluate the normality of the scales’ distributions. We assumed indices less than the ± 2 commonly considered acceptable limits of a normal distribution [74]. No variables exceeded the cutoffs of ± 2.

**Table 1:** Descriptive statistics, normality and reliability tests

Variables	Mean ± SD	Kurtosis	Skewness	Cronbach’s Alpha
(1) Family functioning	6.09 ± 2.61	1.23	0.95	0.89
(2) Hope	26.17 ± 5.84	0.83	1.02	0.91
(3) Resilience	57.81 ± 7.33	1.37	0.86	0.92
(4) Life satisfaction	19.59 ± 4.06	0.74	1.14	0.88

Table 2 presented the bivariate correlations of family functioning, hope, resilience and life satisfaction. A parametric Pearson’s r correlation analysis was performed. As shown in Table 2, family functioning was positively related to hope [r = 0.51, *p* < 0.01], positively related to resilience [r = 0.43, *p* < 0.01], and positively related to students’ life satisfaction [r = 0.59, *p* < 0.01]. Hope was positively related to students’ life satisfaction [r = 0.63, *p* < 0.01]. At the same time, resilience was also positively related to students’ life satisfaction [r = 0.52, *p* < 0.01]. These results provided preliminary supports for the hypotheses proposed above.

**Table 2:** Correlation between for study variables and collinearity statistics

Variables	Correlation coefficient				Collinearity statistics	
	(1)	(2)	(3)	(4)	Tolerance	VIF
(1) Family functioning	1				0.55	3.61
(2) Hope	0.51**	1			0.73	2.59
(3) Resilience	0.43**	0.68**	1		0.69	5.07
(4) Life satisfaction	0.59**	0.63**	0.52**	1	–	–

**p < 0.001

In order to identify multicollinearity, the model was tested because of correlations among all input variables (family functioning, hope, resilience). In the whole sample, the tolerance values ranged from 0.55 to 0.73, above the critical value of 0.1 suggested by Kline [62] as a considerable collinearity problem. The variance inflation factor (VIF) values ranged from 2.59 to 5.07, respectively, not exceeding the commonly assumed threshold of 10.0. Both results suggest that multicollinearity was unlikely to be an issue in our study (Table 2).

In the first step of structural equation modeling analysis, the measurement model was tested. It consisted of two latent variables: hope and resilience. The hope latent variable has two subscales: agency and pathways thinking. The resilience latent variable has five subscales: Personal competence, tolerance of negative emotions, positive acceptance of change, perceived control, and spiritual influences. The measurement model fit was acceptable: x^2^/df = 2.94, CFI = 0.92, TLI = 0.95, SRMR = 0.06; RMSEA = 0.07.

Next, we tested a structural model to examine our hypotheses. The model consisted of one independent variable (family functioning), two mediator variables (hope, resilience), and on dependent variable (life satisfaction). Our hypothesized structural model demonstrated a good fit to our data. Table 3 displays the fitness indices of the hypothesized structural model.

**Table 3:** Fitness indicators of the final hypothesized model

Indices	X^2^/df	SRMR	CFI	TLI	RMSEA
Research model	1.92	0.04	0.95	0.97	0.05
Status	Good	Good	Good	Good	Good

n, 463; CFI, Comparative fit index; TLI, Tucker–Lewis index; RMSEA, Root-mean square error of approximation; SRMR, Standardized root-mean square residual

**Fig. 1:**
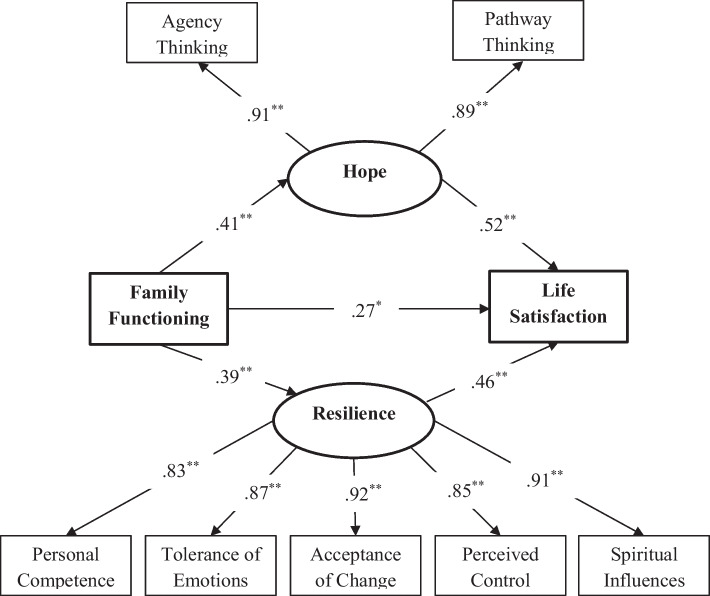
Final mediation model with standardized path coefficients. *p < 0.01; *p < 0.001

The results of the model are shown in Fig. 1. The results of path analysis indicating that family functioning had a statistically significant positive direct effect on hope [β = 0.41, *p* < 0.001], on resilience [β = 0.39, *p* < 0.001] and on life satisfaction [β = 0.27, *p* < 0.01]. The direct path from hope to life satisfaction [β = 0.52, *p* < 0.001] was statistically significant. At the same time, the direct path from resilience [β = 0.46, *p* < 0.001] to life satisfaction was also statistically significant.

In the next step, bias-corrected bootstrapping method was used to test the mediation effect of hope and resilience. The bootstrapping sample size was set to 5,000, the confidence interval was set to 95% and the results were shown in Table 4. The results showed that at the 95% confidence interval level, the indirect effect of hope was 0.21, with a confidence interval of [0.18, 0.39], excluding zero. This indicated that the mediating effect of hope was reliable. The indirect effect of resilience was 0.18, with a confidence interval of [0.13, 0.21], did not include zero. This indicated that the mediating effect of resilience was reliable.

**Table 4:** Standardized indirect path and bootstrapping test

Indirect path	Bootstrapping		BC 95% CI	
	Indirect effect	SE	Lower bound	Upper bound
FF → H → LS	0.21	0.02	0.18	0.39
FF → R → LS	0.18	0.01	0.13	0.21

FF, Family functioning; H, Hope; R, Resilience; LS, Life satisfaction

## Discussion

The COVID-19 pandemic has resulted in a sudden transition from face-to-face teaching and learning to online education in many universities worldwide, such as Lorestan University in Iran. COVID-19 has negatively affected students’ life satisfaction because of its uncertain consequences and the implementation of social distancing measures [6, 15]. Our research expanded earlier reports and assessed the associations between family functioning, hope, resilience, and life satisfaction in female university students during the COVID-19 pandemic in Iran using a mediation model. We found a positive association between family functioning and life satisfaction (Hypothesis 1). These results support previous findings that satisfaction with family ties and a higher familial sense of togetherness, improving life satisfaction in female university students when facing a public health emergency [32, 33]. Family played an important role in female students’ lives during the outbreak due to low accessibility to direct peer support and university support [28]. Desirable family function reflects good relationships and communication between family members, resulting in more positive emotions; it also reflects a lower level of family conflict, helpful for coping with stress effectively and giving more energy to face different things, and consequently maintaining the female students’ life satisfaction in a good state [30]. Families with stronger cohesion can understand family responsibilities and roles and tend to feel more intimate. They are more satisfied and flexible in resolving emotional conflicts [75]. Hence, greater satisfaction with family function increased the life satisfaction of female students during the COVID-19 pandemic.

We revealed that hope could mediate the relationship between family functioning and life satisfaction in female university students, confirming Hypothesis 2, which is in line with previous reports demonstrating hope as a mediator [11, 45]. Hope alleviates the emotions caused by adverse life events, indicating a new perspective for explaining the relationship between family functioning and life satisfaction in female university students. Also, higher hope levels can maintain mental health and increase life satisfaction [34, 35]. Negative emotions of female university students during the COVID-19 pandemic affect their experiences and goals, while family support can ease anxiety and reduce stress. Also, successful coping experiences and positive emotional experiences can effectively motivate female students to solve problems and achieve goals [43], which is consistent with the Circumplex Model of Family Systems [30], indicating that female students with a sense of belonging to friends, family, and relatives could give themselves a great sense of hope [36]. Our findings indicated that female students’ hope could predict their satisfaction with life. Those with a higher sense of hope are less likely to experience depression and anxiety [42]. They also tend to be happier, healthier, and less troubled [39, 41], increasing female students’ satisfaction with life. Therefore, hope mediates the link between female university students’ family functioning and life satisfaction.

Our third hypothesis dealt with the mediating role of resilience in the relationship between female students’ life satisfaction and family functioning, and our results in this regard are in line with other studies [55, 58]. Challenges of COVID-19 confront female students with different psychological reactions, such as fear, sorrow, and stress [20, 21]. Such challenges are various; in the face of life challenges, female students show positive or negative reactions, such as resilience, and such positive reactions provide the foundation for adaptation, fighting problems, and experiencing higher life satisfaction [49]. Also, based on the Circumplex Model of Family Systems, high family adaptability is helpful for female family members in understanding their roles, functions, power, and family expectations, leading to responding effectively to life events. Family members develop more positive competence, like resilience, and become confident through effective coping with events and increasing their capacity to control their environment [57].

Moreover, those with disengaged and chaotic family functioning indicate less adaptive coping strategies and resilience, resulting in lower life satisfaction [33]. Perceiving one’s own family as poor in the parental bond due to a lack of care or over-support can disrupt emotional development, resulting in less functional ways of regulating personal emotions [54]. Parental behaviors, like psychological control, limit the development of personal competencies such as resilience, which can result in depression, anxiety, and mental disorders [56, 58]. Hence, the experience of unbalanced family functioning might make it more problematic to develop a positive model of the self and manage one’s own emotions. Consequently, lower resilience can lead to poorer satisfaction with life [53]. Therefore, resilience mediates the relationship between female university students’ family functioning and life satisfaction.

### Limitations of the study

There are some limitations to the current study that need to be considered. First, this study only recruited female students from one university. Diversified samples should be employed in future studies to enhance the generalizability of the research findings. Second, data was collected through self-report measures, which can influence the validity of the findings. Although the Harmon’ one-factor test was used to verify that there was no serious common method bias, it was not possible to completely exclude the possibility of its existence. Future research can used a staged data collection approach to weaken common method bias. Third, cross-sectional data are used in this study, which makes it difficult to explain the causal relationship between variables. Future studies need to collect longitudinal data to verify the findings of the current study.

### Practical implication

We provided some clinical and practical implications. Our research provides a better perspective regarding the protective effects of family functioning, hope, and resilience on life satisfaction among female university students during the COVID-19 outbreak. Training such skills is needed to increase life satisfaction. Hence, hope and resilience interventions, such as online or face-to-face training, are needed to increase female university students’ hope and resilience levels, promote their capacity to deal with stress problems, enhance positive psychological resources, and establish a preventive mechanism to improve their satisfaction with life. Also, considering the effect of family functioning on the life satisfaction of female university students, we should customize family service interventions to increase effective interactions and communications within the family, provide female students with more material support, improve family relationships and functioning, and increase female students’ life satisfaction.

## Conclusion

The present study contributes to the literature by testing the mediating role of resilience and hope underlying the relationship between family functioning and life satisfaction in Iranian female university students during COVID-19. In conclusion, the results highlighted that family functioning was essential during the COVID-19 outbreak due to its strong relationship with life satisfaction among female university students. Hope is a pathway through which family function increases life satisfaction. The impact of family function on life satisfaction was mediated by resilience. Our research is innovative in assessing resilience and hope as mediators, which had not been assessed by other studies on Iranian female university students. This construct should be more considered among female students, particularly during the COVID-19 outbreak.

## Data Availability

The dataset used and analyzed during the current study are available from the corresponding author on reasonable request.

## References

[1] Hoffart A, Johnson SU, Ebrahimi OV (2020). Loneliness and social distancing during the COVID-19 pandemic: risk factors and associations with psychopathology. Front Psychiatry.

[2] Gawrych M, Cichoń E, Kiejna A (2021). COVID-19 pandemic fear, life satisfaction and mental health at the initial stage of the pandemic in the largest cities in Poland. Psychol Health Med.

[3] Lipskaya-Velikovsky L (2021). COVID-19 isolation in healthy population in Israel: challenges in daily life, mental health, resilience, and quality of life. Int J Environ Res Public Health..

[4] Lee J (2020). Mental health effects of school closures during COVID-19. Lancet Child Adolesc Health.

[5] United Nations Educational SaCOU. Covid-19 educational disruption and response. Unesco. 2020.

[6] Sahu P (2020). Closure of Universities due to Coronavirus Disease 2019 (COVID-19): impact on Education and Mental Health of students and academic staff. Cureus.

[7] Pavot W, Diener E (2008). The satisfaction with life scale and the emerging construct of life satisfaction. J Posit Psychol.

[8] Diener E (2012). New findings and future directions for subjective well-being research. Am Psychol.

[9] López-Ortega M, Torres-Castro S, Rosas-Carrasco O (2016). Psychometric properties of the satisfaction with life scale (SWLS): secondary analysis of the Mexican health and aging study. Health Qual Life Outcomes.

[10] Yang C, Xia M, Han M, Liang Y (2018). Social support and resilience as mediators between stress and life satisfaction among people with substance use disorder in China. Front Psychiatry.

[11] Wider W, Taib NM, Khadri M, Yip FY, Lajuma S, Punniamoorthy PA (2022). The unique role of hope and optimism in the relationship between environmental quality and life satisfaction during COVID-19 pandemic. Int J Environ Res Public Health..

[12] Cacioppo M, Pace U, Zappulla C (2013). Parental psychological control, quality of family context and life satisfaction among italian adolescents. Child Indic Res.

[13] Gomez-Baya D, Muñoz-Silva A, Garcia-Moro FJ (2020). Family climate and life satisfaction in 12-year-old adolescents in Europe. Sustainability.

[14] Kleszczewska D, Dzielska A, Salonna F, Mazur J (2018). The association between physical activity and general life satisfaction in lower secondary school students: the role of individual and family factors. Community Ment Health J.

[15] Evli M, Şimşek N (2022). The effect of COVID-19 uncertainty on internet addiction, happiness and life satisfaction in adolescents. Arch Psychiatr Nurs.

[16] Gigantesco A, Fagnani C, Toccaceli V, Stazi MA, Lucidi F, Violani C (2019). The relationship between satisfaction with life and depression symptoms by gender. Front Psychiatry.

[17] Lee J, Kim E, Wachholtz A (2016). The effect of perceived stress on life satisfaction: the mediating effect of self-efficacy. Chongsonyonhak Yongu.

[18] Cao W, Fang Z, Hou G, Han M, Xu X, Dong J (2020). The psychological impact of the COVID-19 epidemic on college students in China. Psychiatry Res.

[19] Rogowska AM, Kuśnierz C, Bokszczanin A, Examining Anxiety L, Satisfaction (2020). General health, stress and coping styles during COVID-19 pandemic in polish sample of university students. Psychol Res Behav Manag.

[20] Chinna K, Sundarasen S, Khoshaim HB, Kamaludin K, Nurunnabi M, Baloch GM (2021). Psychological impact of COVID-19 and lock down measures: an online cross-sectional multicounty study on asian university students. PLoS One.

[21] Lopes AR, Nihei OK (2021). Depression, anxiety and stress symptoms in brazilian university students during the COVID-19 pandemic: predictors and association with life satisfaction, psychological well-being and coping strategies. PLoS One.

[22] Özmen S, Özkan O, Özer Ö, Yanardağ MZ (2021). Investigation of COVID-19 fear, well-being and life satisfaction in turkish society. Soc Work Public Health.

[23] Tekir Ö (2022). The relationship between fear of COVID-19, psychological well-being and life satisfaction in nursing students: a cross-sectional study. PLoS One.

[24] Bambra C, Albani V, Franklin P (2021). COVID-19 and the gender health paradox. Scand J Public Health.

[25] Solomou I, Constantinidou F (2020). Prevalence and predictors of anxiety and depression symptoms during the COVID-19 pandemic and compliance with precautionary measures: age and sex matter. Int J Environ Res Public Health..

[26] Zhang Y, Zhang H, Ma X, Di Q (2020). Mental health problems during the COVID-19 pandemics and the mitigation effects of exercise: a longitudinal study of college students in China. Int J Environ Res Public Health.

[27] Prowse R, Sherratt F, Abizaid A, Gabrys RL, Hellemans KGC, Patterson ZR (2021). Coping with the COVID-19 pandemic: examining gender differences in stress and mental health among university students. Front Psychiatry.

[28] Szcześniak M, Tułecka M (2020). Family functioning and life satisfaction: the mediatory role of emotional intelligence. Psychol Res Behav Manag.

[29] Minuchin S, Rosman BL, Baker L. Cambridge. MA and London, England: Harvard University Press; 2013.

[30] Olson DH (2000). Circumplex model of marital and family systems. J Fam Ther.

[31] Olson DH, Waldvogel L, Schlieff M (2019). Circumplex model of marital and family systems: an update. J Fam Ther Rev.

[32] Wenzel K, Townsend J, Hawkins B, Russell B (2020). Changes in family leisure functioning following a family camp for children with autism spectrum disorder (ASD). Ther Recreat J.

[33] Huang Z, Zhang L, Wang J, Xu L, Wang T, Tang Y (2022). Family function and life satisfaction of postgraduate medical students during the COVID-19 pandemic: the mediating role of meaning in life and depression. Heliyon.

[34] Bailey TC, Eng W, Frisch MB, Snyder CR (2007). Hope and optimism as related to life satisfaction. J Posit Psychol.

[35] Yang Y, Zhang M, Kou Y (2016). Self-compassion and life satisfaction: the mediating role of hope. Pers Individ Differ.

[36] Fredrickson BL (2001). The role of positive emotions in positive psychology. The broaden-and-build theory of positive emotions. Am Psychol.

[37] Peter C, Müller R, Cieza A, Post MW, van Leeuwen CM, Werner CS (2014). Modeling life satisfaction in spinal cord injury: the role of psychological resources. Qual Life Res.

[38] Snyder CR (2002). Hope theory: rainbows in the mind. Psychol Inq.

[39] Gungor A, Avci M, editors. Examining the relationship between hope and life satisfaction among middle school students; 2017.

[40] Karataş Z, Uzun K, Tagay Ö (2021). Relationships between the life satisfaction, meaning in life, hope and COVID-19 fear for Turkish adults during the COVID-19 outbreak. Front Psychol.

[41] Marques SC, Lopez SJ, Mitchell J (2013). The role of hope, spirituality and religious practice in adolescents’ life satisfaction: longitudinal findings. J Happiness Stud.

[42] Connelly TW (2005). Family functioning and hope in children with juvenile rheumatoid arthritis. MCN Am J Matern Child Nurs.

[43] Song Y, Cui C, Jia Y, Zhang W, Meng L, Sznajder KK (2022). Family functioning and optimism as protective factors of life satisfaction among stroke patients during the COVID-19 epidemic in Shenyang, China. Front Public Health.

[44] Liu C, Cheng Y, Hsu ASC, Chen C, Liu J, Yu G (2018). Optimism and self-efficacy mediate the association between shyness and subjective well-being among Chinese working adults. PLoS One.

[45] Yun P, Xiaohong H, Zhongping Y, Zhujun Z, Family Function (2021). Loneliness, emotion regulation, and hope in secondary vocational school students: a moderated mediation model. Front Public Health.

[46] Kumpfer K, Glantz M, Johnson J (1999). Factors and processes contributing to resilience: the resilience framework. Resilience and development: positive life adaptations.

[47] Russo SJ, Murrough JW, Han MH, Charney DS, Nestler EJ (2012). Neurobiology of resilience. Nat Neurosci.

[48] Shi M, Wang X, Bian Y, Wang L (2015). The mediating role of resilience in the relationship between stress and life satisfaction among Chinese medical students: a cross-sectional study. BMC Med Educ.

[49] Liang S, Dong M, Zhao H, Song Y, Yang A (2022). Mindfulness and life satisfaction: the moderating effect of self-control and the moderated moderating effect of resilience. Pers Individ Differ.

[50] Cohn MA, Fredrickson BL, Brown SL, Mikels JA, Conway AM (2009). Happiness unpacked: positive emotions increase life satisfaction by building resilience. Emotion.

[51] Kjeldstadli K, Tyssen R, Finset A, Hem E, Gude T, Gronvold NT (2006). Life satisfaction and resilience in medical school—a six-year longitudinal, nationwide and comparative study. BMC Med Educ.

[52] Prayag G, Spector S, Orchiston C, Chowdhury M (2020). Psychological resilience, organizational resilience and life satisfaction in tourism firms: insights from the Canterbury earthquakes. Curr Issues Tour.

[53] Baykal E (2020). Effects of resilience on life satisfaction among employees during COVID-19 pandemic. J Cyprus Stud.

[54] Hu J, Ye B, Yildirim M, Yang Q (2022). Perceived stress and life satisfaction during COVID-19 pandemic: the mediating role of social adaptation and the moderating role of emotional resilience. Psychol Health Med.

[55] Kukihara H, Yamawaki N, Ando M, Nishio M, Kimura H, Tamura Y (2020). The mediating effect of resilience between family functioning and mental well-being in hemodialysis patients in Japan: a cross-sectional design. Health Qual Life Outcomes.

[56] Leys C, Kotsou I, Goemanne M, Fossion P (2017). The influence of family dynamics on eating disorders and their consequence on resilience: a mediation model. Am J Fam Ther.

[57] Nam B, Kim JY, DeVylder JE, Song A (2016). Family functioning, resilience, and depression among North Korean refugees. Psychiatry Res.

[58] Ng YY, Sulaiman WSW (2017). Resilience as mediator in the relationship between family functioning and depression among adolescents from single parent families. Akademika.

[59] Michalski CA, Diemert LM, Hurst M, Goel V, Rosella LC (2022). Is life satisfaction associated with future mental health service use? An observational population-based cohort study. BMJ Open.

[60] Antaramian S (2017). The importance of very high life satisfaction for students’ academic success. Cogent Educ.

[61] Jensen P, Haug E, Sivertsen B, Skogen JC (2021). Satisfaction with life, mental health problems and potential alcohol-related problems among Norwegian university students. Front Psychiatry.

[62] Kline R (2011). Principles and practice of structural equation modeling.

[63] Diener E, Emmons RA, Larsen RJ, Griffin S (1985). The satisfaction with life scale. J Pers Assess.

[64] Motevaliyan SM, Dokoushkani F, Yahyazadeh Jeloudar S (2019). Study of life satisfaction among students of University of Mazandaran and its relationship with personality dimensions. Shenakht J Psychol Psychiatry.

[65] Smilkstein G (1978). The family APGAR: a proposal for a family function test and its use by physicians. J Fam Pract.

[66] Smilkstein G, Ashworth C, Montano D (1982). Validity and reliability of the family APGAR as a test of family function. J Fam Pract.

[67] Kahrazee F, Rigi Kooteh B (2016). Relationship between the family function with academic self-regulation among the nursing students. B Educ Strategy Med Sci.

[68] Snyder CR, Sympson SC, Ybasco FC, Borders TF, Babyak MA, Higgins RL (1996). Development and validation of the state hope scale. J Pers Soc Psychol.

[69] Zarei S, Fooladvand K (2019). A mediating role of hope in the relationship between self-compassion and life satisfaction; a non-interventional study. Health Res J.

[70] Connor KM, Davidson JR (2003). Development of a new resilience scale: the Connor–Davidson resilience scale (CD-RISC). Depress Anxiety.

[71] Sadoughi M (2018). The relationship between academic self-efficacy, academic resilience, academic adjustment, and academic performance among medical students. B Educ Strategy Med Sci.

[72] Lt Hu, Bentler PM (1999). Cutoff criteria for fit indexes in covariance structure analysis: conventional criteria versus new alternatives. Struct Equ Model.

[73] Podsakoff PM, MacKenzie SB, Lee JY, Podsakoff NP (2003). Common method biases in behavioral research: a critical review of the literature and recommended remedies. J Appl Psychol.

[74] Gravetter F, Wallnau L (2014). Essentials of statistics for the behavioral sciences.

[75] Zhang Y (2018). Family functioning in the context of an adult family member with illness: a concept analysis. J Clin Nurs.

